# Surface Decarburization Depth Detection in Rods of 60Si2Mn Steel with Magnetic Barkhausen Noise Technique

**DOI:** 10.3390/s23010503

**Published:** 2023-01-02

**Authors:** Peng Li, Xianxian Wang, Dongdong Ding, Zhaoxiang Gao, Wen Fang, Chaolei Zhang, Cunfu He, Xiucheng Liu

**Affiliations:** 1 Faculty of Information Technology, Beijing University of Technology, Beijing 100124, China; 2 School of Materials Science and Engineering, University of Science and Technology Beijing, Beijing 100083, China

**Keywords:** magnetic Barkhausen noise, surface decarburization depth, core microstructure, spring steel

## Abstract

Magnetic Barkhausen noise (MBN), sensitive to the microstructure of materials, can be applied in the surface decarburization depth detection of ferromagnetic specimens. However, the effects of core microstructures on the determination results of decarburization depth have not been explored. In this study, MBN was employed to evaluate the magnetic properties of the decarburized 60Si2Mn spring steels with martensitic and pearlitic core microstructures. Spring steel samples were austenitized at different times to generate different decarburization depths. Seven magnetic features were extracted from the MBN butterfly profiles. We used the variation coefficient, linear correlation coefficient, and normalized sensitivity to discuss the influence of the core microstructures on these seven features. The different core microstructures led to a large difference in the ability of MBN features to characterize the decarburization layer depth. However, three features of MBN butterfly profiles demonstrated an approximately linear dependency (linear correlation coefficient > 94%) on surface decarburization depth and monotonically increased with the increase in depth in both core microstructures of spring steels.

## 1. Introduction

In the heat treatment process of steels, they are often required to be heated to the austenitizing temperature, which easily leads to the loss of carbon and the formation of a surface decarburized layer [1]. The formed decarburized layer changes the surface microstructure and has undesirable effects on some physical and mechanical properties of steel, such as hardness, wear, and fatigue resistance [2]. Therefore, the accurate measurement of decarburization depth is important in the quality assessment of steel production lines.

Common decarburization detection methods, such as metallographic analysis or microhardness measurement, are destructive, time-consuming, and cannot be applied in online processing. Over the past ten years, the feasibility of measuring decarburization with nondestructive testing (NDT) methods has been investigated, such as eddy current (EC) [3,4,5], magnetic hysteresis curve [6,7], and magnetic Barkhausen noise (MBN) [8,9,10]. A ferrite layer on a ferrite-70% pearlite structure in AISI 1055 steel was determined with a normalized impedance of EC signals. With the increase in decarburization depth, both magnetic permeability and normalized impedance output increased [5]. A similar microstructural change had also been detected using the MBN technique [5]. Kahrobaee et al. measured the decarburization depth of Hadfield steels with three NDT methods and found that magnetic flux leakage had a better linear relationship with decarburization depth compared with the magnetic hysteresis curve and EC method [7]. Among those NDT methods, the MBN technique is sensitive to surface microstructure [11] and contains lots of magnetic features [12]; therefore, it is a promising candidate for the characterization of the decarburization depth. Saquet et al. compared the MBN signals from different microstructures (ferrite, pearlite, and martensite) of plain carbon steels and found that the shape, amplitude, and position of the MBN were strongly influenced by the microstructure [13]. Blaow et al. [8] found that the MBN profile of the decarburized Ovako 677 steel sample had a double-peak profile and that the height and position of the second peak were related to the decarburization depth. A similar double-peak phenomenon had been reported in isothermally annealed samples of low-carbon 18CrNiMo5 and high-carbon 42CrMo4 steels with MBN measurement [14]. However, the effects of core microstructures on the decarburization detection ability of MBN technology have not been studied.

In this paper, the MBN technique was employed to evaluate the decarburization depth of 60Si2Mn steel rods of different core microstructures. Several features of MBN butterfly profiles could be correlated with decarburization depth, and the correlations between MBN features and decarburization depth remained unchanged under different core microstructures. In summary, MBN technology can be used to determine decarburization depth.

## 2. Materials and Methods

### 2.1. Sample Preparation

Common spring steel (60Si2Mn) with a high content of carbon (Table 1) was chosen for the experiment because its high strength was strongly influenced by decarburization. Samples were heat treated by austenitizing at 750 °C, followed by air cooling. Two microstructures, such as pearlite and martensite in the core of steel, were produced by changing the cooling rate of austenite.

Fourteen steel rods of 100 mm in length and 10 mm in diameter (Figure S1 in the Supporting Information) were prepared from one base material for the decarburization process. One rod was used as-received without any decarburization layer and showed the mixed microstructure of ferrite and pearlite (Figure S2 in the Supporting Information). Eight rods with martensitic core microstructure were austenitized at 750 °C for 0.5, 1, 1.5, 2, 3, 4, 5, and 6 h to generate different decarburization depths. The other five rods with pearlitic core microstructures were austenitized at 750 °C for 0.25, 1, 4, 5, and 6 h. Four evenly-spaced positions around the cross-section of each rod were marked for MBN measurements and subsequent metallographic experiments, considering the slight difference in decarburization layer depth. After the MBN measurements of all samples, the microstructures and decarburized layers of the decarburized samples were studied with a metallographic microscope (LEXT OLS4100, OLYMPUS). Table 2 and Table 3 summarize the collected data of complete decarburization depth obtained in metallographic experiments of all the samples.

### 2.2. MBN Tests

**Figure 1:**
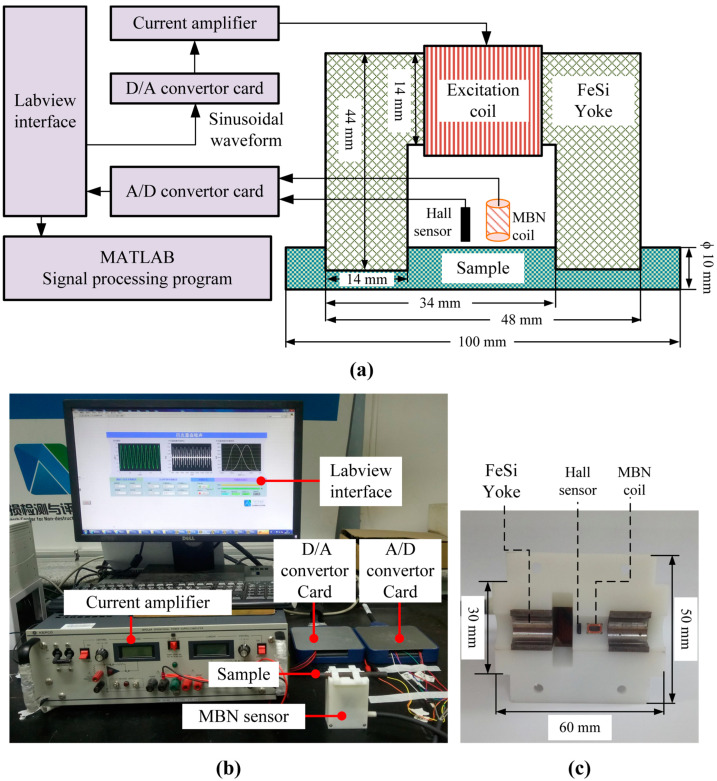
(**a**) Schematic diagram (not to scale), photograph (**b**) of the experimental set-up for MBN measurements, and (**c**) cross-section of the MBN sensor.

A special MBN sensor was designed for rod samples. The schematic illustration and photograph of the MBN system are shown in Figure 1. The MBN system is mainly composed of D/A and A/D convertor cards, a bipolar current amplifier, an MBN sensor, a Labview (Version 2014, National Instruments Corporation) interface, and the MATLAB (Version R2020b, MathWorks.Inc.) analysis program.

An MBN sensor consists of a FeSi yoke, excitation and MBN coils, a hall sensor, and an external support structure. In order to magnetize the steel rod, 450 turns of enameled wire with a diameter of 0.38 mm (named excitation coils) were wound on the middle arm of the U-shaped FeSi yoke. A sinusoidal signal (50 Hz, 3 Vpp) was generated by the D/A convertor card (PXIe 6376, National Instruments Corporation ), further amplified by a bipolar current amplifier (BOP 100-4DL, KEPCO.INC), and finally applied at excitation coils. A hall sensor (SS39E, HONEYWELL) and MBN coil (400 turns of enameled wire with a diameter of 0.05 mm) 0.5 mm above the steel surface were used to measure the tangential magnetic field (TMF) and MBN signals, respectively. The high-speed A/D convertor card (PXIe 6376, National Instruments Corporation ) collected the TMF and MBN signals at a sampling rate of 1 M/s with an accuracy of 16 bits. The D/A and A/D convertor cards were controlled by a Labview interface. The collected data were further processed by MATLAB analysis program. The MBN butterfly profiles were plotted with TMF and MBN signals. Several magnetic features of the MBN butterfly profiles were extracted to characterize the thickness of the decarburized layer.

## 3. Results

### 3.1. Measurements of Sample Decarburization Depth

**Figure 2:**
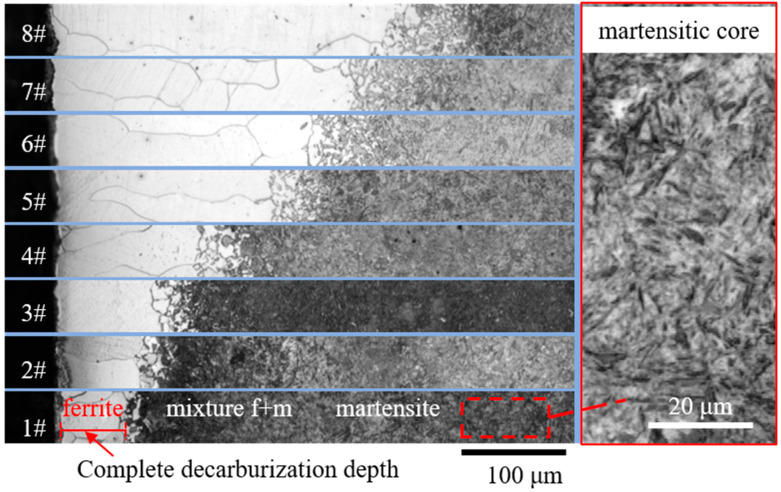
Metallographic pictures of the cross-sections of steel samples with martensitic core austenitized for 0.5 h (Sample 1#), 1 h (Sample 2#), 1.5 h (Sample 3#), 2 h (Sample 4#), 3 h (Sample 5#), 4 h (Sample 6#), 5 h (Sample 7#), and 6 h (Sample 8#). The needle-like microstructure can be observed in the martensitic core.

**Figure 3:**
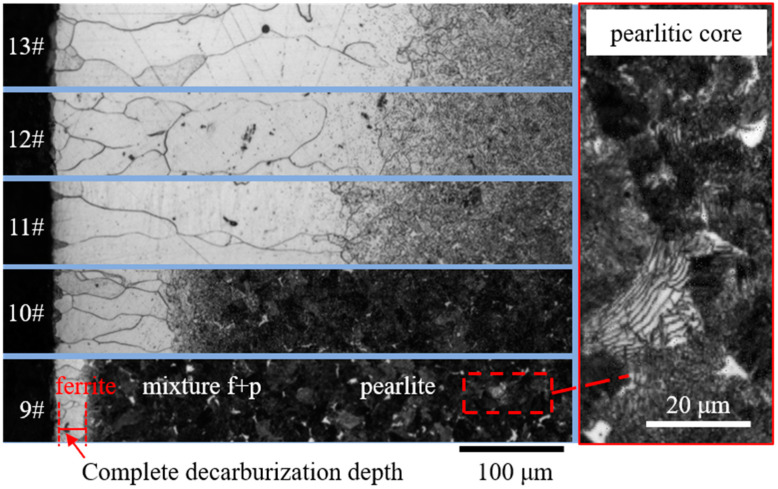
Metallographic pictures of the cross-sections of steel samples with pearlitic core austenitized for 0.25 h (Sample 9#), 1 h (Sample 10#), 4 h (Sample 11#), 5 h (Sample 12#), and 6 h (Sample 13#). The layer-like microstructure can be seen in the pearlitic core.

**Figure 4:**
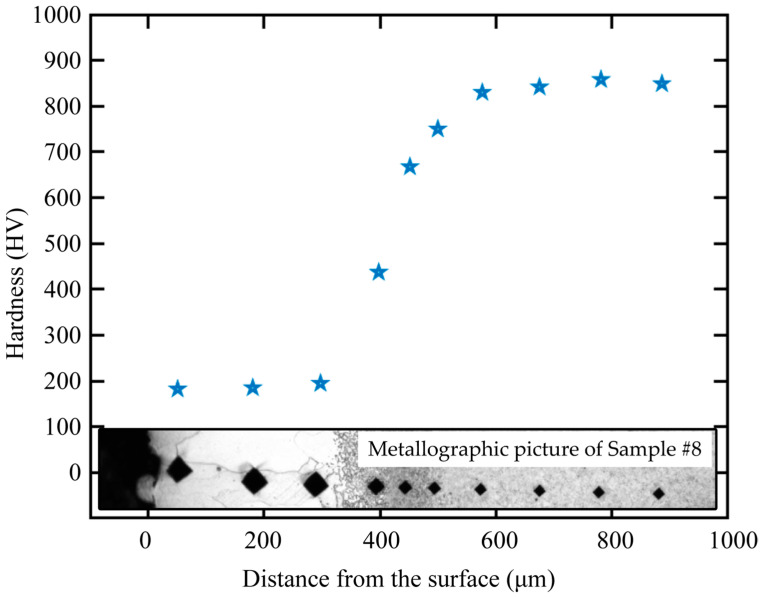
Hardness profiles and metallographic picture of the steel sample with a martensitic core austenitized for 6 h (Sample 8#).

**Figure 5:**
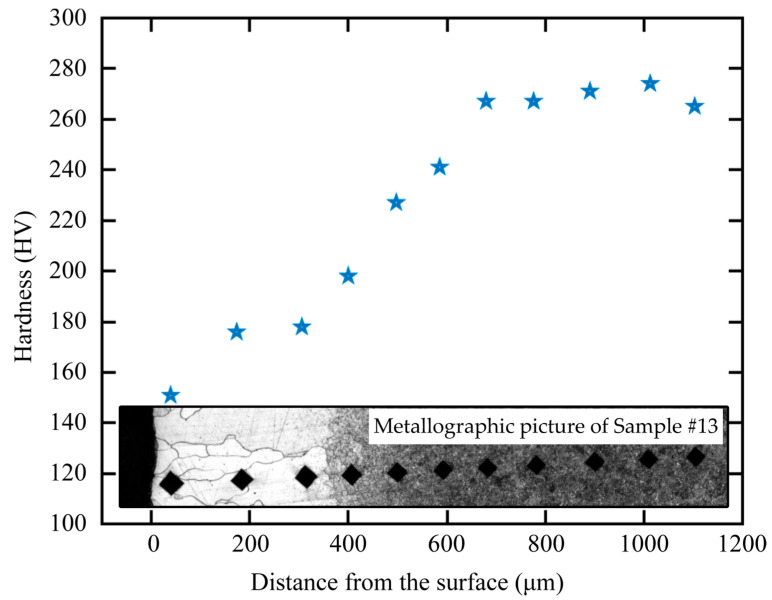
Hardness profiles and metallographic picture of the steel sample with a pearlitic core austenitized for 6 h (Sample 13#).

**Figure 6:**
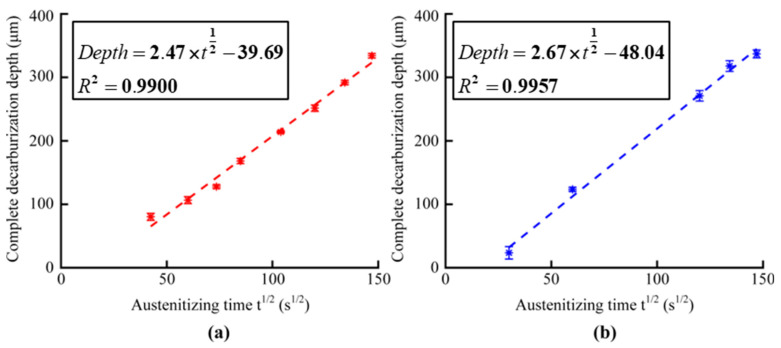
Complete decarburization depth of samples with an (**a**) martensitic core and (**b**) pearlitic core, depending on austenitizing treatment time. Four positions of each sample were measured.

Figure 2 and Figure 3 show the metallographic pictures of spring steels with different core microstructures decarburized for different austenitizing times. After austenitizing, a thin layer with a clear boundary around the core microstructure appeared on the surface of spring steel. In the thin layer, the grain size was relatively large, indicating a single ferrite microstructure. A needle-like microstructure could be observed (Figure 2), indicating that the core microstructure was a martensite microstructure [15]. The microstructure evolution of the decarburization layer was also revealed by the hardness profile (Figure 4). Decarburization produced a ferritic microstructure with a hardness of about 180 HV, which was lower than the bulk hardness of the martensite (850 HV). The layered microstructure indicated that the core microstructure (Figure 3) was pearlite. The hardness was also about 180 HV at the thin ferrite layer, which gradually increased in the decarburization layer and reached 270 HV at the pearlitic core (Figure 5). The thickness of the decarburization layer significantly increased with the increase in austenitizing time (Figure 2 and Figure 3). The thin ferrite layer is defined as complete decarburization, whose depth can be accurately measured with image processing software (Image-Pro Plus, Media Cybernetics.). The complete decarburization depth at four positions for each sample was measured (Table 2 and Table 3). The relationships between complete decarburization depth and austenitizing treatment time at 750 °C are given in Figure 6. It was found that the complete decarburization depth of the sample with martensitic and pearlitic core microstructures was a function of the square root of the austenitizing time, as reported by other researchers [16].

### 3.2. MBN Feature Selection

Multiple experiments were conducted with all samples to check the repeatability of MBN signal testing. During the experiments, four evenly-spaced positions around the cross-section of each rod were marked, and MBN measurements in every position of the rods were conducted six times. After all of the samples were tested, a total of (6 × 4 × 13 =) 312 sets of MBN signals were obtained.

**Figure 7:**
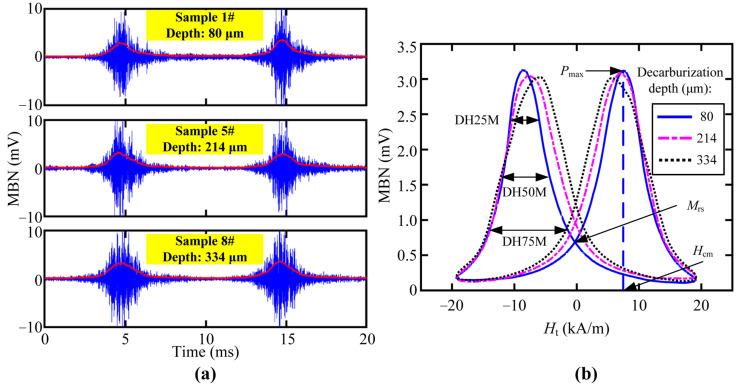
MBN test of the samples with a martensitic core: (**a**) waveforms of the MBN signals obtained from the samples with different decarburization depths and (**b**) calculated MBN butterfly profiles of the samples with different decarburization depths.

**Figure 8:**
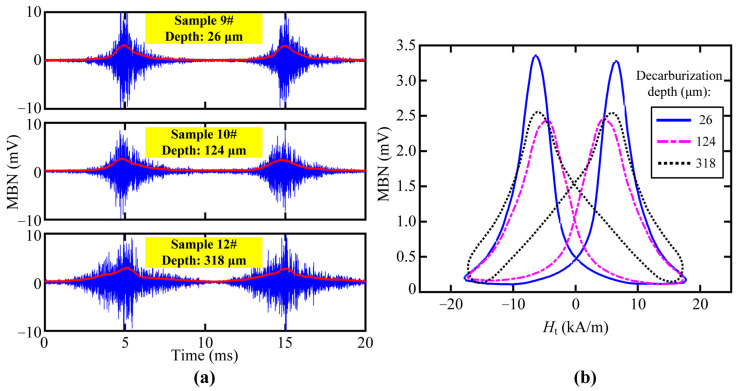
MBN test of the samples with a pearlitic core: (**a**) waveforms of the MBN signals obtained from the samples with different decarburization depths and (**b**) calculated MBN butterfly profiles of the samples with different decarburization depths.

A sinusoidal magnetizing current with a frequency of 50 Hz was fed into the excitation coil during the tests. The typical measured MBN signal waveforms filtered by a four-order Butterworth band-passed filter (20–120 kHz) are shown in Figure 7 and Figure 8. The time domain signals (blue lines in Figure 7a and Figure 8a) were processed by sliding average (sliding step of 800 points) so as to obtain the MBN envelopes (red lines in Figure 7a and Figure 8a), whose dependency on the TMF strength (*H*_t_) could be plotted as the butterfly profile (Figure 7b and Figure 8b). For each measurement, the smoothed MBN butterfly profiles in three magnetizing cycles were averaged to minimize the error. The shape of the butterfly profile was affected by the depth of the decarburized layer, and thus several candidate features, such as the height or peak position of the butterfly profile, could be extracted for the characterization of decarburized layer thickness.

Common candidate features, including peak height *P*_max_, peak position *H*_cm_, or 75% height of width DH75M, were extracted from the MBN butterfly profiles; the seven total features are summarized in Table 4, which are also reported in our previous work [17]. For each candidate feature, the variation coefficient δ for the repeated six measurements was evaluated. The variation coefficient δ is defined as:

(1) $$\delta =\frac{\sigma }{\bar{X}}$$

where σ and $\bar{X}$ are the standard deviation and mean value of the candidate feature, respectively. Then, the average value of δ for all the samples ($\delta_{a}$) was used as the criteria for the feature selection. Table 4 gives the statistical results of the coefficient of variation of the nine micromagnetic features. Among those nine features, *H*_cm_ and DH75M had a large value of *δ* ($\delta_{a}$ > 5%), indicating that the repeated test data showed significant dispersion and might not be suitable for subsequent quantitative analysis.

## 4. Discussion

A simple linear fitting method was used to analyze the relationship between the seven magnetic features and the depth of the surface decarburized layer. The fitting correlation coefficient (*R*^2^) and normalized sensitivity per depth (ξ) are calculated for each feature (Table 5). Normalized sensitivity per depth ξ is defined as

(2) $$\xi =\frac{\Delta X}{X_{\max}\Delta D}$$

where ΔD is the change in depth, ΔX is the change in the candidate magnetic feature, and $X_{\max}$ is the maximum value of the candidate magnetic feature.

**Figure 9:**
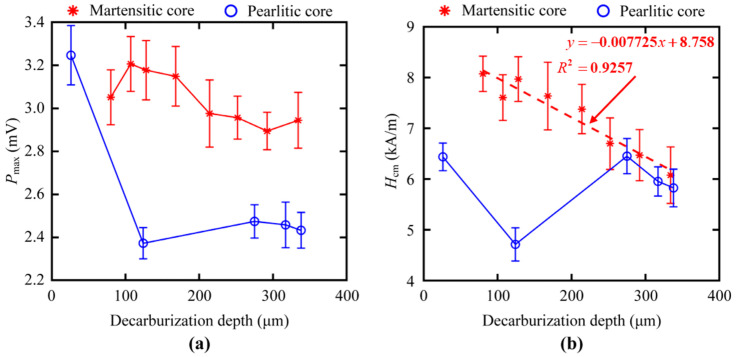
Dependency of features (**a**) *P*_max_ and (**b**) *H*_cm_ on the surface decarburization depths for all samples with martensitic and pearlitic cores.

*P*_max_ had a relatively low value of *R*^2^ in both core microstructures of samples, indicating the non-linearity dependency on the sample decarburization depth (Figure 9a). Each data point was a mean value of 24 identical measurements (six measurements × four positions). The error bars present the corresponding standard error. Ferrite microstructure had fewer total pinning agents and equivalent unpinning instances than both martensite and pearlite microstructures; therefore, the *P*_max_ value of MBN decreased with the increase in the ferrite microstructure of the decarburized layer (Figure 9a), which was similar to the results reported by Stupakov et al. [10].

The relationship between the *H*_cm_ and depth in samples with martensitic core and pearlite is drawn in Figure 9b. Ferrite has smaller coercivity than pearlite and martensite [18]. Consequently, the *H*_cm_ value representing the coercive field (red line in Figure 9b) decreased with the increase in the decarburization depth of the samples with a martensitic core. This can also be explained by the relationship between the coercivity field and mechanical hardness. When the martensite volume fraction decreases with the increases in the decarburization depth of the martensitic core samples, the coercive field decreases with the decrease in the mechanical hardness [19]. In addition, the *H*_cm_ (blue line in Figure 9b) obtained from the samples with pearlitic core showed a similar decrease with the increase in the large decarburization depth, but it exhibited a non-monotonous dependency at the beginning. This discrepancy at the thinner decarburized layer might be partly ascribed to residual stress in the thinner decarburized layer on the pearlitic core.

**Figure 10:**
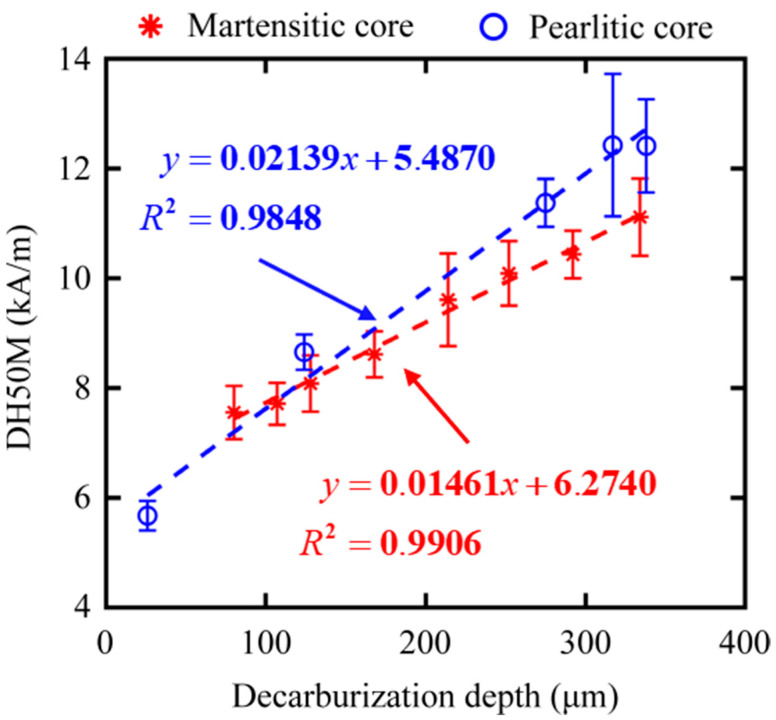
Dependencies of feature DH50M on the surface decarburization depths for all samples with martensitic and pearlitic cores.

**Figure 11:**
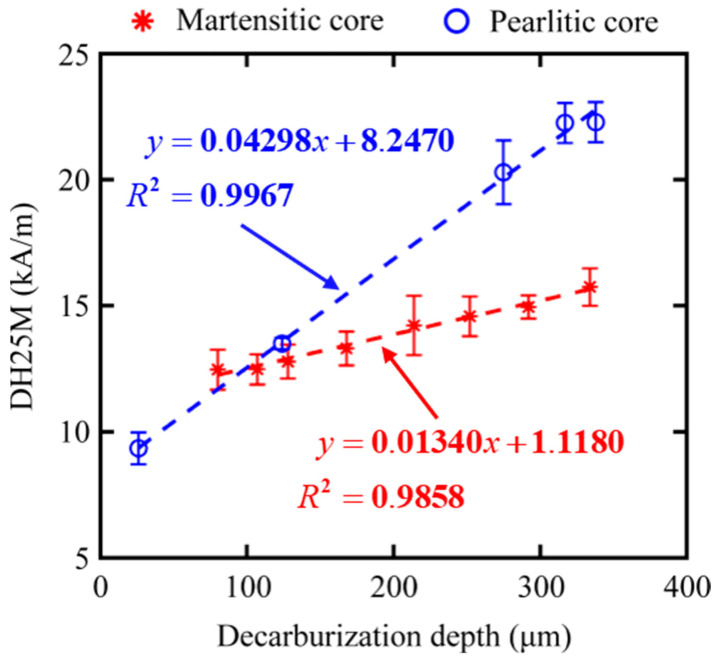
Dependencies of feature DH25M on the surface decarburization depths for all samples with martensitic and pearlitic cores.

**Figure 12:**
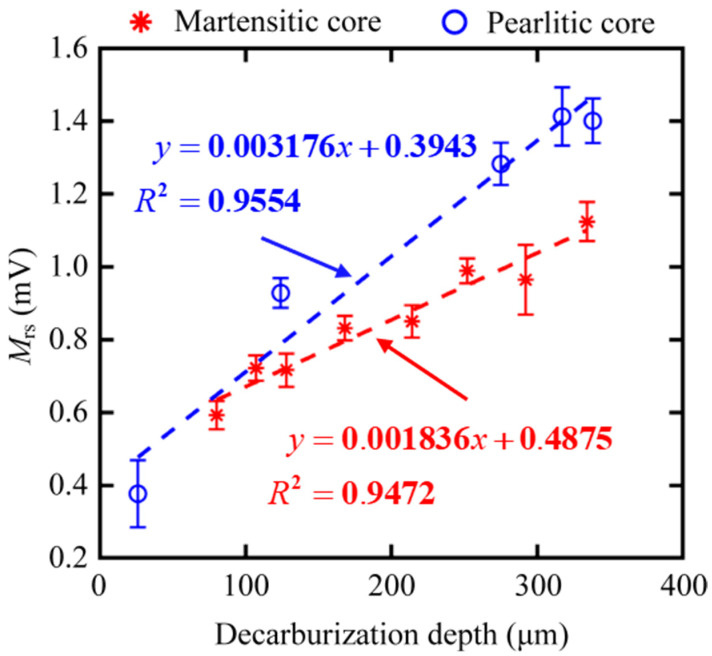
Dependencies of feature *M*_rs_ on the surface decarburization depths for all samples with martensitic and pearlitic cores.

From other features, we preferably selected three magnetic features, such as DH50M, DH25M, and *M*_rs_, with good *R*^2^ in both core microstructures of samples. The dependencies of feature DH50M, DH25M, and *M*_rs_ on the surface decarburization depths for all the samples are shown in Figure 10, Figure 11 and Figure 12, respectively. The three magnetic features were linearly correlated with the depth, and they showed the same trend under the different core microstructures. In other words, the three magnetic features of MBN could be used to characterize the decarburization depth, which had less influence on core microstructures. Different microstructures were observed in the samples with decarburization (Figure 2 and Figure 3), as indicated by multiple peaks (or the merging of multiple peaks) in the MBN butterfly profiles [14]. The multiple peak phenomena demonstrated by the MBN signal of Sample 12# (shown in Figure 8b) might be responsible for why the peak width (presented with DH50M and DH25M features) increased with the increase in decarburization depth. In addition, we further used ξ to represent the sensitivity of the preferred magnetic feature to depth. The ξ of DH50M, DH25M, and *M*_rs_ had larger values in the samples with martensitic core than those in the samples with pearlitic core (Table 5).

## 5. Conclusions

In this paper, the ability of MBN to nondestructively characterize the decarburization depth of spring steel was investigated, and the effects of different core microstructures on the characterization ability of MBN were explored. The main results are summarized as follows:

1. Firstly, heat treatment of spring steels with two different core microstructures, such as martensitic and pearlitic cores, resulted in decarburization and the formation of a thin layer on the surface of the steel sample. The metallographic results confirmed that the core microstructures to ferrite transformation occurred on the surface after heat treatment. The depth of complete decarburized layers in both core microstructures of samples was linearly related to the square root of the heat treatment time.
2. In the nondestructive assessment of the decarburization depth with MBN in both core microstructures of the samples, it was found that the microstructural changes of the decarburization layer had significant changes on the magnetic properties and induced the change in MBN butterfly profiles. Therefore, MBN signals could be used to determine the occurrence of the decarburization layer and quantify the decarburization depth.
3. Seven magnetic features were extracted from the MBN butterfly profiles. The influence of the core microstructures on these seven features was discussed. The seven MBN features performed differently with the core microstructures. However, three MBN magnetic features, such as DH50M (*R*^2^ > 0.98), DH25M (*R*^2^ > 0.98), and *M*_rs_ (*R*^2^ > 0.94), were obtained as the optimum output and showed a monotonic increase trend with depth in both core microstructure types of samples.
4. For those optimum features of MBN, the variation coefficient, linear correlation coefficient, and normalized sensitivity were different between the two core microstructures.

## Figures and Tables

**Table 1:** Chemical composition of steel 60Si2Mn.

Steel	C	Si	Mn	P	S	Cr	Ni	Cu	Al	Ti
60Si2Mn	0.58	1.70	0.80	0.016	0.004	0.23	0.01	0.01	0.02	0.01

**Table 2:** Complete decarburization depths estimated based on metallographic analysis of all samples with martensitic core microstructure.

Sample Nos.	Austenitizing Time (h)	Complete Decarburization Depth (μm)				
		Position 1	Position 2	Position 3	Position 4	Average
1	0.5	85	75	76	85	80
2	1	112	110	100	104	107
3	1.5	126	132	132	126	128
4	2	164	165	165	172	168
5	3	215	213	213	212	214
6	4	245	256	256	255	252
7	5	287	292	292	293	292
8	6	336	331	331	338	334

**Table 3:** Complete decarburization depths estimated based on metallographic analysis of all samples with pearlitic core microstructure.

Sample Nos.	Austenitizing Time (h)	Complete Decarburization Depth (μm)				
		Position 1	Position 2	Position 3	Position 4	Average
9	0.25	-	15	32	32	26
10	1	120	128	123	-	124
11	4	276	272	277	259	275
12	5	310	313	329	319	318
13	6	336	332	346	334	338

**Table 4:** Features extracted from the typically measured MBN butterfly profiles.

Indicators	Units	Descriptions	$\mathrm{Martensitic}\;\mathrm{Core}\;\delta_{a}$	$\mathrm{Pearlitic}\;\mathrm{Core}\;\delta_{a}$
*P*_max_	mV	Peak height of the MBN butterfly curve	3.14%	2.94%
*H*_cm_	kA/m	Peak position of MBN butterfly curve	4.24%	5.37%
DH75M	kA/m	75% Height width	2.84%	5.08%
DH50M	kA/m	50% Height width	2.82%	4.28%
DH25M	kA/m	25% Height width	1.77%	2.21%
*M*_rs_	mV	Amplitude at remanent point	3.97%	3.65%
*P*_mean_	mV	Amplitude averaged over one magnetization cycle	2.01%	1.67%

**Table 5:** Correlation coefficient and normalized sensitivity per depth of features extracted from the typically measured MBN profiles.

Indicators	Martensitic Core		Pearlitic Core	
	*R* ^2^	*ξ* (%/μm)	*R* ^2^	*ξ* (%/μm)
*P*_max_	0.6492	-	0.5321	-
*H*_cm_	0.9257	0.097	0.0088	-
DH75M	0.9829	0.12	0.8277	0.15
DH50M	0.9906	0.13	0.9848	0.21
DH25M	0.9858	0.082	0.9967	0.23
*M*_rs_	0.9472	0.19	0.9554	0.29
*P*_mean_	0.7823	0.052	0.9234	0.12

## Data Availability

Not applicable.

## Supplementary Materials

The following are available online at https://www.mdpi.com/article/10.3390/s23010503/s1, Figure S1: Photograph of spring steel rods, Figure S2: Metallographic picture of the as- received steel sample without any decarburized layer.
