# Supplementary material for: Surface Decarburization Depth Detection in Rods of 60Si2Mn Steel with Magnetic Barkhausen Noise Technique
Source: Sensors (Basel). 2023 Jan 2;23(1):503. doi: 10.3390/s23010503 (PMC9823766; doi:10.3390/s23010503)
Supplement: Supplementary file 1 [file sensors-23-00503-s001.zip › sensors-2125772-supplementary.pdf]

Supporting Information

Surface Decarburization Depth Detection in Rods of 60Si2Mn Steel with Magnetic Barkhausen Noise Technique

Peng Li [1], Xianxian Wang [1], Dongdong Ding [1], ZhaoXiang Gao [1], Wen Fang [2], Chaolei Zhang [2], Cunfu He [1], and Xiucheng Liu[1, *]

[1] Faculty of Information Technology, Beijing University of Technology, Beijing 100124, China

[2] School of Materials Science and Engineering, University of Science and Technology Beijing, Beijing 100083, China

* Correspondence: xiuchliu@bjut.edu.cn; Tel.: +86-010-6739-1720

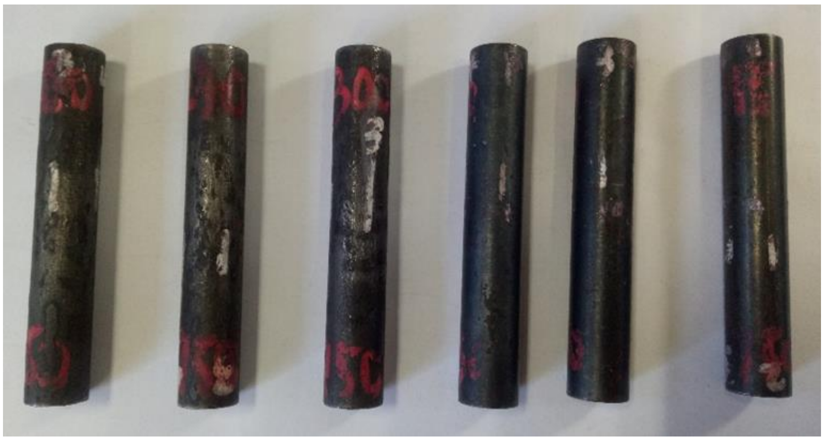

Figure S1. Photograph of spring steel rods.

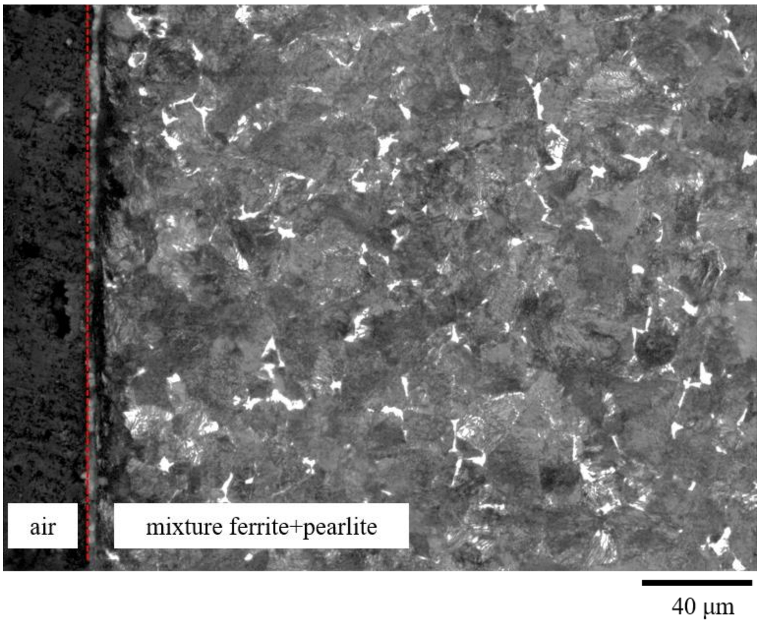

Figure S2. Metallographic picture of the as- received steel sample without any decarburized layer.
